# Systematic Assessment of the Climate Sensitivity of Important Human and Domestic Animals Pathogens in Europe

**DOI:** 10.1038/s41598-017-06948-9

**Published:** 2017-08-02

**Authors:** K. Marie McIntyre, Christian Setzkorn, Philip J. Hepworth, Serge Morand, Andrew P. Morse, Matthew Baylis

**Affiliations:** 1 Department of Epidemiology and Population Health, Institute of Infection and Global Health, University of Liverpool, Leahurst Campus, Neston, Cheshire CH64 7TE UK; 2 NIHR Health Protection Research Unit in Emerging and Zoonotic Infections, Liverpool, L69 7BE UK; 3 CNRS ISEM - CIRAD ASTRE, Faculty of Veterinary Technology, Kasetsart University, Bangkok, Thailand; 4 Department of Helminthology, Faculty of Tropical Medicine, Mahidol University, Bangkok, 10400 Thailand; 5 Department of Geography and Planning, School of Environmental Sciences, Roxby Building, University of Liverpool, Liverpool, L69 7ZT UK

## Abstract

Climate change is expected to threaten human health and well-being via its effects on climate-sensitive infectious diseases, potentially changing their spatial distributions, affecting annual/seasonal cycles, or altering disease incidence and severity. Climate sensitivity of pathogens is a key indicator that diseases might respond to climate change, but the proportion of pathogens that is climate-sensitive, and their characteristics, are not known. The climate sensitivity of European human and domestic animal infectious pathogens, and the characteristics associated with sensitivity, were assessed systematically in terms of selection of pathogens and choice of literature reviewed. Sixty-three percent (N = 157) of pathogens were climate sensitive; 82% to primary drivers such as rainfall and temperature. Protozoa and helminths, vector-borne, foodborne, soilborne and waterborne transmission routes were associated with larger numbers of climate drivers. Zoonotic pathogens were more climate sensitive than human- or animal-only pathogens. Thirty-seven percent of disability-adjusted-life-years arise from human infectious diseases that are sensitive to primary climate drivers. These results help prioritize surveillance for pathogens that may respond to climate change. Although this study identifies a high degree of climate sensitivity among important pathogens, their response to climate change will be dependent on the nature of their association with climate drivers and impacts of other drivers.

## Introduction

Climate change is expected to threaten human health and well-being via its effects on climate-sensitive infectious diseases, potentially changing their spatial distributions, affecting annual or seasonal cycles, altering disease incidence and in some cases disease severity^1^. There is a growing body of evidence suggesting that climate change is already driving certain diseases to higher latitudes^2, 3^ or altitudes^4^, and the expectation must be that such effects will continue, and perhaps accelerate in future, as the global climate continues to warm and rainfall patterns change. But while some diseases appear to be responding to climate change, many do not^5^.

This raises important questions: how large is the threat posed by climate change across the full range of diseases that affect us? Will the majority of diseases respond to climate change or only a few? What are the characteristics of those diseases that will respond? Is it possible that the most significant infectious diseases in health or economic terms could be resilient, so that the overall influence of changes in climate could be of relatively minor importance for infectious disease spread^1^? The answers to these questions are likely to influence the policy debate and the scale of interventions including adaptation and mitigation measures^6^.

Numerous studies have examined the effects of climate change upon specific diseases, mostly using modelling approaches because the long time scale over which changes in climate occur does not favour empirical approaches. Modelling approaches include statistical modelling^2, 7, 8^, ecological niche modelling^9^, biological, process-based modelling^3, 10^, climate simulation-based risk modelling^11, 12^ or meta-analytical approaches using electronic data-mining^13^. In addition, the relative impact of climate has been examined for certain diseases or restricted sets of diseases using qualitative and semi-quantitative risk assessment based methods^14–16^. In one study, Lindgren and others^17^ identified a direct or indirect link between climate and almost half of a set of human notifiable, haemorrhagic or emerging diseases in Europe, based upon a systematic literature review coupled with expert judgment. No studies, however, have used fully systematic approaches to assess the scale of the climate change impact on the range of diseases that significantly impact on human health, and there have been no systematic reviews of the impact on animal health.

Here, we develop a bottom-up, quantitative, risk assessment approach to systematically select high impact pathogens of humans and animals, and review their climate-disease literature, to quantify the sensitivity of the disease burden of people and domestic animals in Europe to climate^18^. In practical terms, it is not feasible to assess the sensitivity to climate of our entire infectious disease burden as estimates of the number of pathogens believed to be infectious to humans range from 1415^19^ to 1752^20^, some with very little clinical effect. Our approach is to focus on those pathogens that have the largest impact on human and domestic animal health^21^, on the assumption that this will capture a large proportion of the overall bearing of climate on health status as a result of infectious diseases.

Ideally the direction of association between a pathogen and its climate drivers would be captured, for example, that higher temperatures will mean pathogen spread or increased incidence. In practice, however, this is rarely possible as relationships are often non-linear and pathogen/disease levels are frequently maximized at intermediate levels of a driver; or the precise relationship to a driver may co-depend on other drivers. Modelling approaches (eg, refs 10 and 22) are therefore required to ascertain the impact of change in climate drivers on the level or transmission risk of a pathogen. Here, therefore, we restrict ourselves to identifying those pathogens that are sensitive to climate drivers, and the characteristics of those pathogens.

## Results

One hundred human and one hundred domestic animal, high-impact, pathogens present in Europe (157 in total, as 43 were in both sets) were systematically reviewed for evidence of sensitivity to 190 climate driver terms, grouped into eleven sets (hereafter referred to as climate drivers). The selection criteria for high impact pathogens and climate drivers are described in the Supplementary Information. Climate drivers were classified as either primary (climate change, climate oscillations, extreme weather events, moisture, rainfall, temperature and wind) or secondary (altitude, vegetation, particulate matter and salinity). Over sixty-three percent of pathogens (99/157) had positive evidence (defined as positive median scores; see Supplementary Information) of links suggesting sensitivity to at least one climate driver; if examining human and animal pathogens separately, numbers are very similar (68% and 70%, respectively) (see Table S6). Nearly thirty-seven percent of pathogens (58/157) lacked positive evidence of a link to climate.

**Figure 1:**
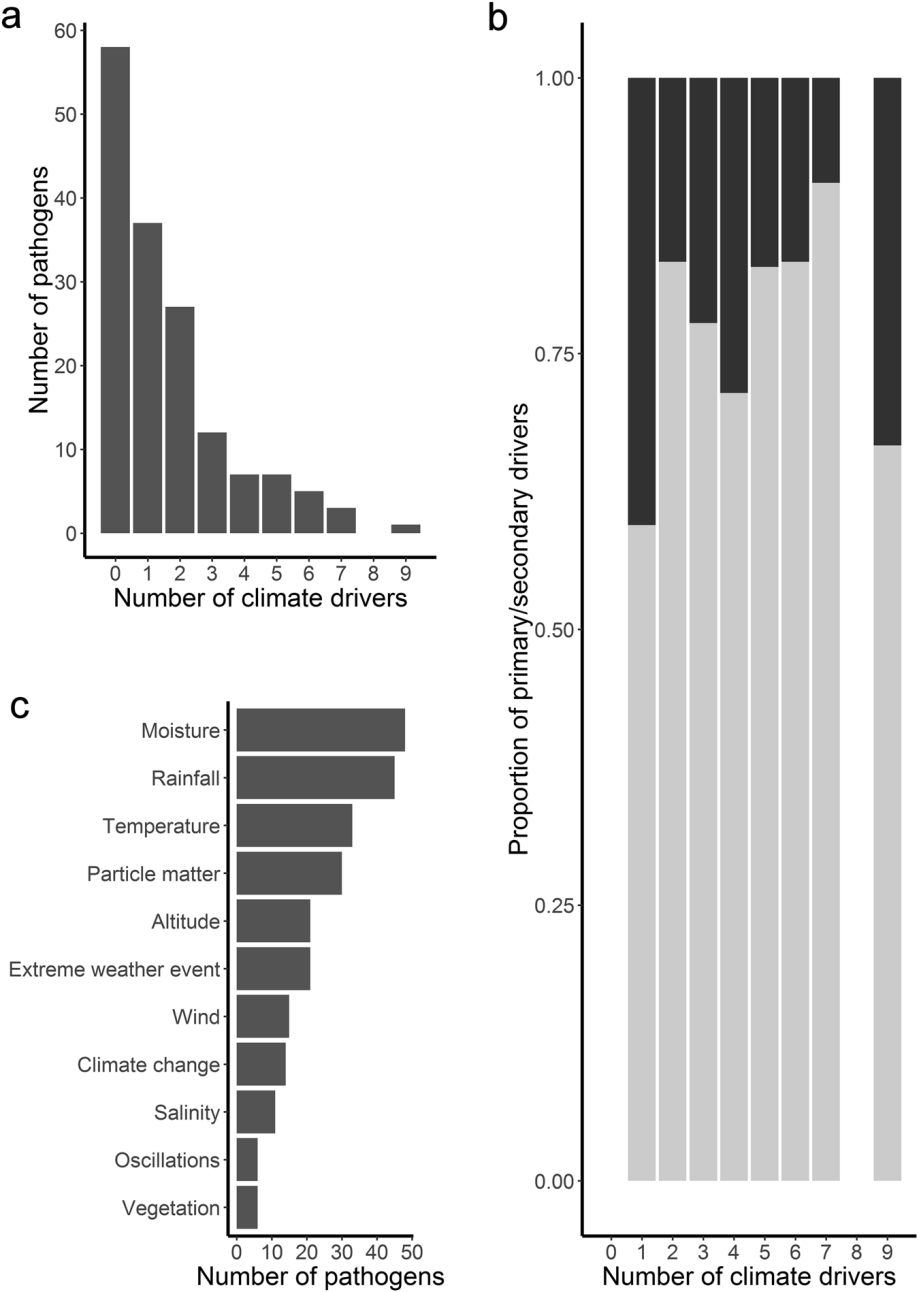
Frequency histograms describing associations between human and domestic animal, high impact pathogens and climate drivers: (**a**) Frequency of pathogens (across all taxa) associated with different numbers of climate drivers, (**b**) Proportion of primary (in dark grey) compared to secondary (in light grey) drivers associated with different numbers of climate drivers, and (**c**) Frequency of pathogens associated with specific climate drivers.

Considering the 99 pathogens with positive evidence for sensitivity to climate drivers, 37.4% had one climate driver and 39.4% had 2 or 3 drivers (Fig. 1a). Over 90% had between 1 and 5 drivers; few (9.1%) had links to more than 5 drivers. Of the 99 pathogens, 81.8% (81/99) had primary climate drivers, while 56.6% (56/99) had secondary drivers. Eighteen pathogens had secondary but not primary climate drivers. The proportion of primary compared to secondary drivers appears to change positively with the number of drivers identified (Fig. 1b); however differences were not statistically significant. The primary or secondary drivers most frequently associated with pathogens were moisture (48/99), rainfall (45/99), temperature (33/99) and particulate matter (30/99) (Fig. 1c). Oscillations and vegetation were the climate drivers least frequently associated with pathogens. Certain climate drivers commonly occurred in combination; the most common being moisture and rainfall, 25.3% (25/99); moisture and temperature, 21.2% (21/99); rainfall and temperature, 18.2% (18/99); and rainfall and extreme weather events, 17.2% (17/99).

**Figure 2:**
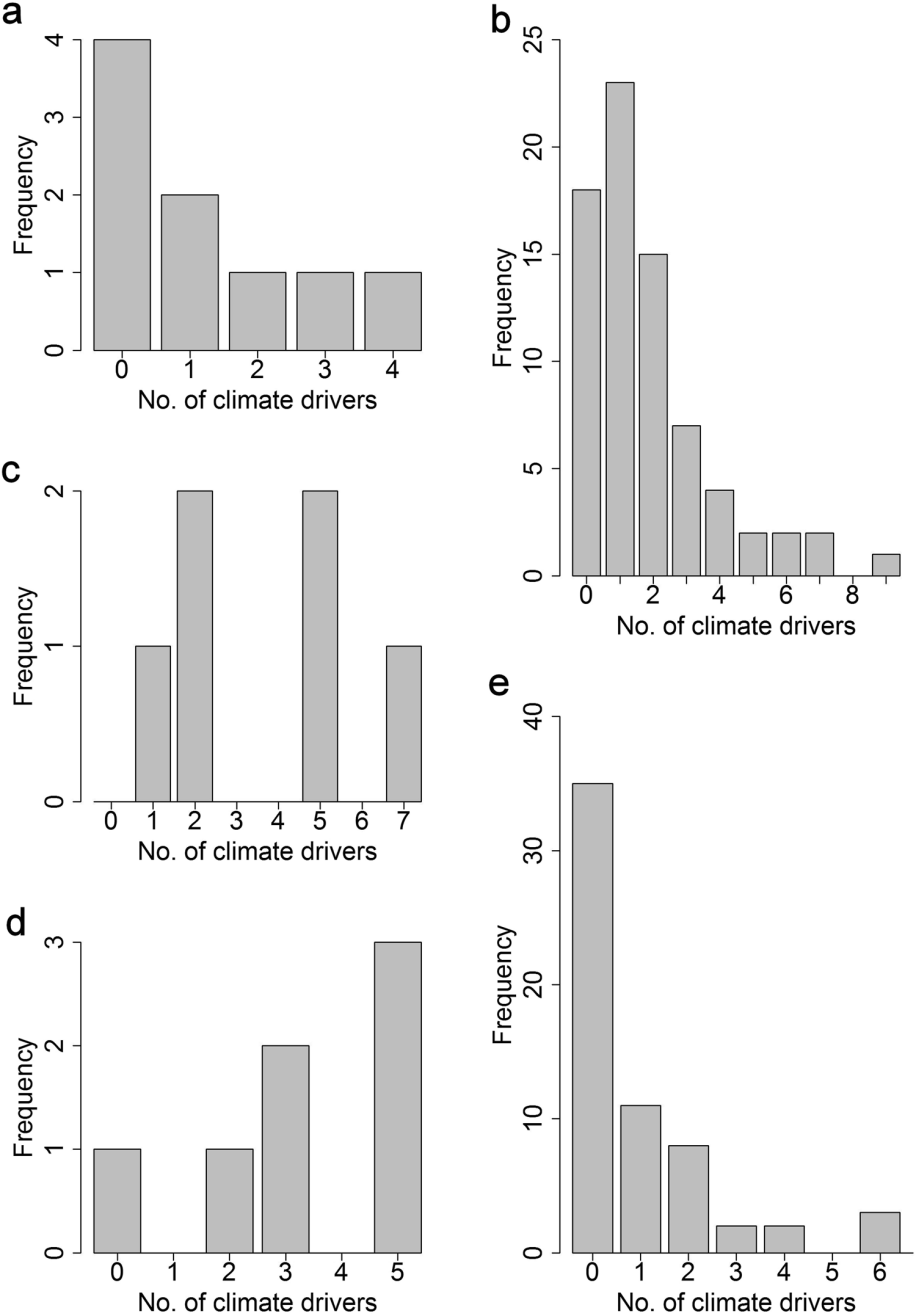
Frequency histograms of human and domestic animal, high impact pathogens associated with different numbers of climate drivers. Bars show the number of (**a**) fungi, (**b**) bacteria, (**c**) helminths, (**d**) protozoa and (**e**) viruses that were identified with 0–9 climate drivers.

There were statistically significant differences among the five major taxonomic divisions of pathogens in the frequency of climate drivers (Table S7). The distribution of the number of climate drivers was right-skewed for bacteria, fungi and viruses (Fig. 2b,a and e, respectively); such that most had few or no drivers and only a small number had many drivers. By contrast, the distributions for protozoa (N = 7) and helminths (N = 6) were left-skewed or bell-shaped (Fig. 2c,d, respectively). Although the numbers examined are small, we found no helminths which lack evidence of climate drivers. The largest number of climate drivers (nine) for an individual pathogen species was for the bacterium *Vibrio cholerae* (the causative agent of cholera). The helminth *Fasciola hepatica* (cause of liver fluke) and the bacteria *Bacillus anthracis* (cause of anthrax) and *Borrelia burgdorferi* (cause of tickborne Lyme disease) each had seven climate drivers.

**Figure 3:**
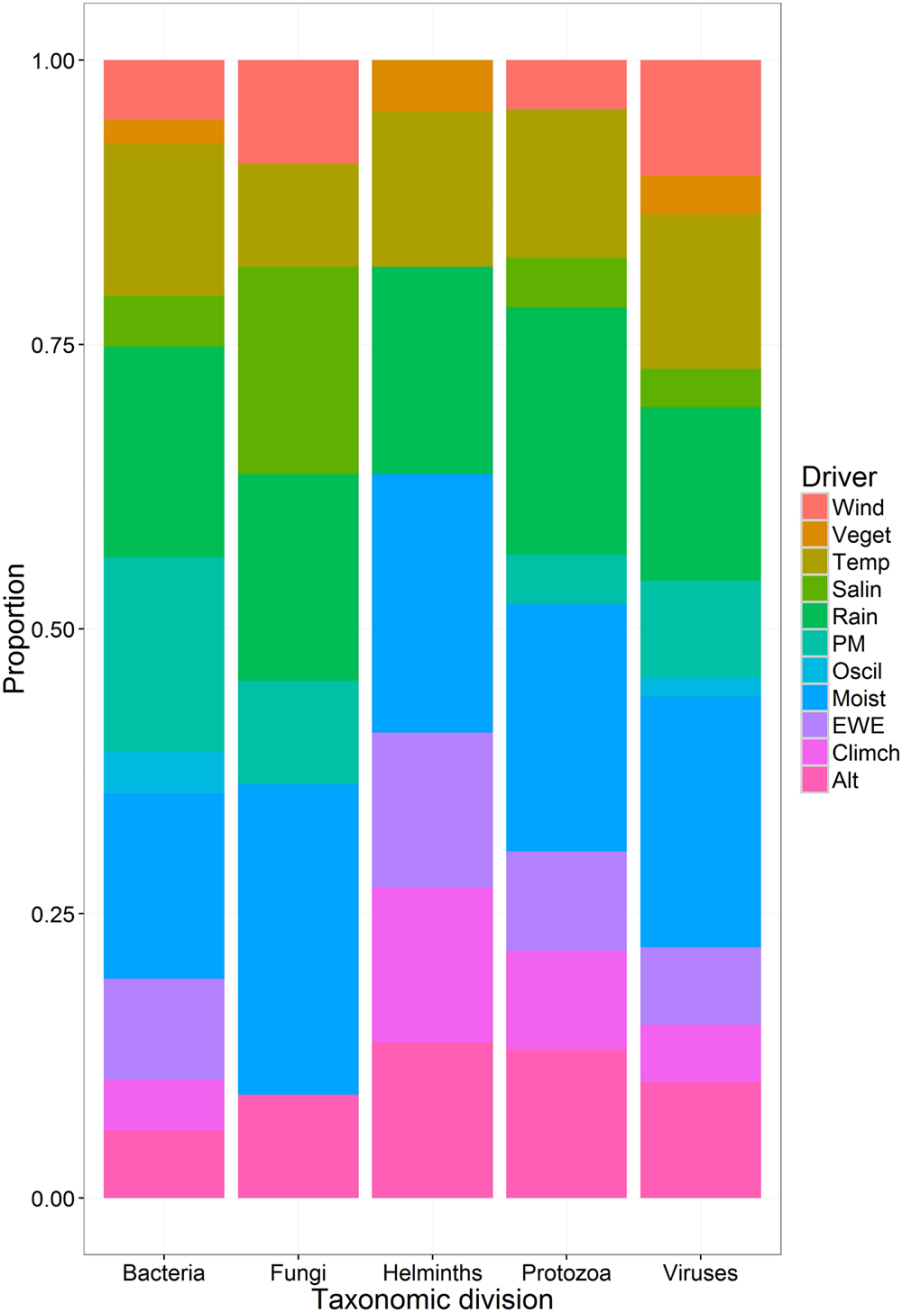
Stacked bar plot showing the proportion of high impact human and domestic animal pathogens sensitive to different climate drivers, broken down by pathogen taxa. Climate drivers are: altitude (Alt), climate change (Climch), extreme weather events (EWE), moisture (Moist), oscillations (Oscil), particulate matter (PM), rainfall (Rain), salinity (Salin), temperature (Temp), vegetation (Veget) and wind (Wind). Note: more than one climate driver can be associated with a pathogen.

Moisture, rainfall and temperature were frequent climate drivers for all five taxonomic groups. Fungi were particularly sensitive to moisture and salinity, and were associated more strongly with wind than other groups except viruses (Fig. 3). Helminths and protozoa were most sensitive to altitude.

**Figure 4:**
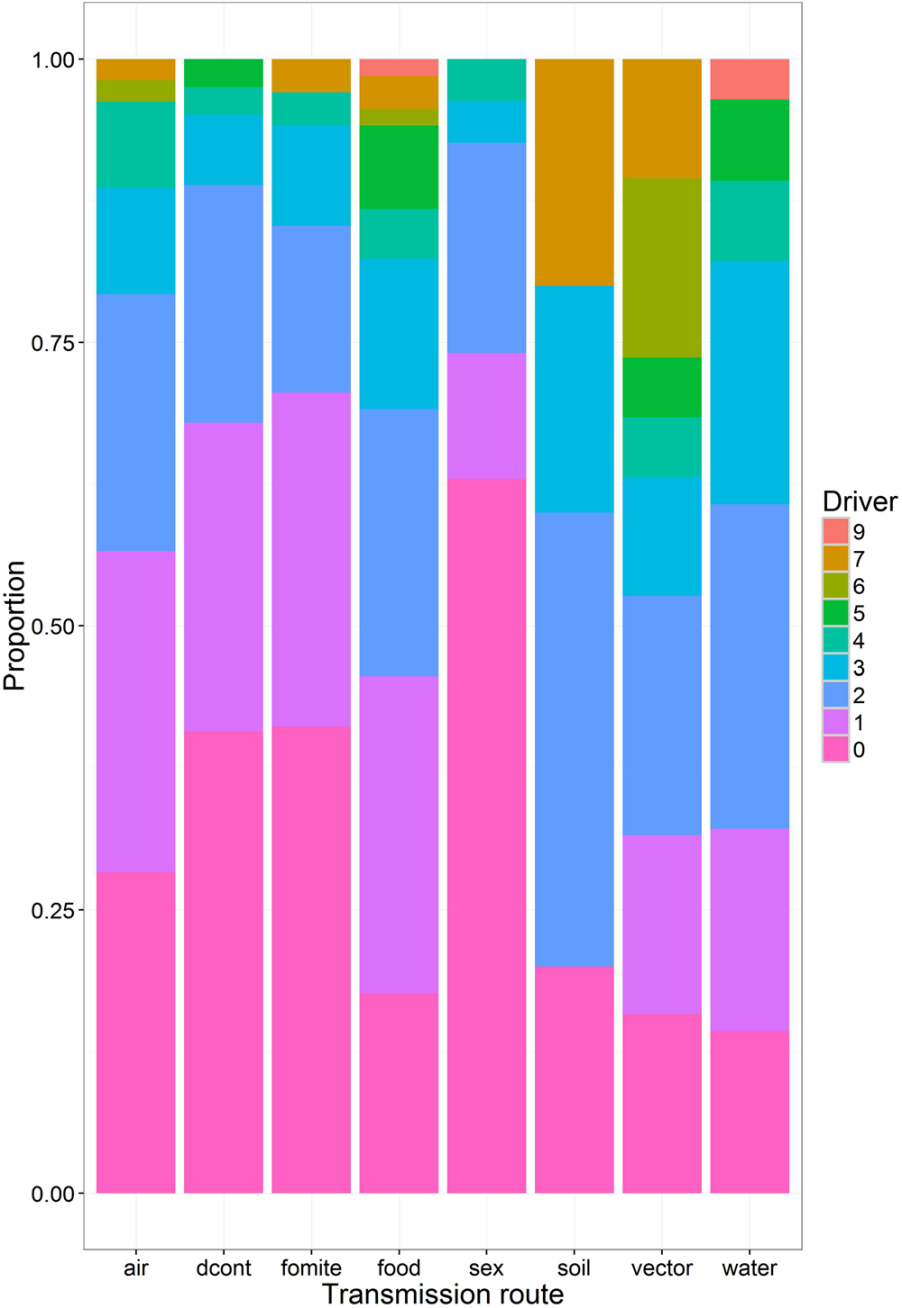
Stacked bar plot showing the proportion of high impact human and animal pathogens with 0–9 climate drivers, broken down by pathogen transmission route. Transmission routes include: environment/airborne (via inhalation) - person (air), nonsexual direct contact (dcont), environment/fomite - person (fomite), foodborne - person (via ingestion) (food), sexual direct contact (sex), environment/soil - person (soil), vector-borne - person (vector) and environment/waterborne - person (water). Note: more than one transmission route can be associated with a pathogen.

The transmission routes for the 157 pathogens were, in decreasing order of frequency: direct (non-sexual) contact (81), foodborne (68), airborne (53), fomite (34), waterborne (28), direct sexual contact (27), vector-borne (19), and soil-borne (5). There were noteworthy associations between transmission route and the frequency of climate drivers (Fig. 4). Vector-borne, soil-borne, waterborne and foodborne pathogens were the most likely to have one or more climate drivers, and pathogens transmitted by direct sexual and non-sexual contact or fomite transmission routes were least likely to have a climate driver. Transmission route was also associated with the number of climate drivers identified, for example over 47% of vector-borne pathogens had three or more climate drivers, compared to 40% for soil-borne, 39% for waterborne and nearly 31% for foodborne. These rankings remain largely the same when four, five or more climate drivers were examined instead. There was no association between the number of transmission routes and the number of climate drivers (Pearson’s product-moment correlation (r) = 0.038, *P* = 0.639), nor associations for primary (r = 0.045, *P* = 0.576) or secondary (r = 0.005, *P* = 0.949) drivers associated with climate.

Pathogens with more climate drivers tended to be reported in a larger number of European countries (*b* = 1.11, *F*_1,155_ = 9.89, *P* = 0.002).

There was some evidence that zoonotic pathogens are more sensitive to climate than those not zoonotic (Table 1). Zoonotic pathogens were more likely than human-only or animal-only pathogens to have at least one climate driver (Pearson’s χ^2^ (1, N = 156) = 22.38, P < 0.001, effect size (ø) = 0.38, odds ratios (ORs) for zoonotic versus human-only or animal-only pathogens were 6.21 and 4.02, respectively). Zoonotic pathogens were also associated with certain transmission routes: foodborne and waterborne pathogens were significantly more likely to be zoonotic, while those that are directly (non-sexually) transmitted were significantly less likely to be zoonotic. If included concurrently in a multivariable model, however, the effects of waterborne transmission were no longer significant. Other transmission routes were not statistically associated with being zoonotic.

**Table 1:** Logistic regression models describing the risk of pathogens being zoonotic compared to non-zoonotic when linked to transmission routes including direct (non-sexual) contact, waterborne and foodborne.

Attribute			95% CL	
		**AOR** ^a^	**Lower**	**Upper**
Univariable direct contact				
No evidence		Baseline	—	—
Positive evidence		**0.24**	0.10	0.59
Univariable waterborne				
No evidence		Baseline	—	—
Positive evidence		**3.19**	1.21	8.38
Univariable foodborne				
No evidence		Baseline	—	—
Positive evidence		**3.25**	1.65	6.43
Multivariable				
No evidence of direct contact		Baseline	—	—
Positive evidence of direct contact		**0.30**	0.12	0.77
No evidence of foodborne		Baseline	—	—
Positive evidence of foodborne		**2.75**	1.37	5.55

^a^AOR, adjusted odds ratios shown in bold differ significantly (P < 0.05) from one.

Specific climate drivers were associated with pathogen emergence (Table 2). Pathogens with rain or climate change as drivers had greater odds of being classed as emerging. The highest (4^th^ quartile) H-index pathogens also had greater odds of being classed as emerging. However, there was no difference between emerging and non-emerging pathogens in the odds of having at least one climate driver (Pearson’s χ^2^ (1, N = 157) = 0.329, *P* = 0.566).

**Table 2:** Logistic regression models describing the risk of pathogens being emerging compared to not emerging when linked to certain climate drivers.

Attribute		AOR^a^	95% CL	
			Lower	Upper
Rain				
	No evidence	Baseline	—	—
	Positive evidence	**2.75**	1.21	6.24
H-indices	1^st^ quartile	Baseline	—	—
	2^nd^ quartile	0.85	0.34	2.11
	3^rd^ quartile	1.24	0.50	3.08
	4^th^ quartile	**3.66**	1.29	10.39
Climate change				
	No evidence	Baseline	—	—
	Positive evidence	3.88^†^	0.81	18.62
H-indices	1^st^ quartile	Baseline	—	—
	2^nd^ quartile	1.04	0.43	2.55
	3^rd^ quartile	1.31	0.53	3.23
	4^th^ quartile	**4.15**	1.47	11.72

^a^AOR, adjusted odds ratios shown in bold differ significantly (P < 0.05) from one;

^†^relationship of borderline significance (*P* = 0.090).

### Climate sensitivity of diseases in Global Burden of Disease (GBD) study

The most comprehensive and recent estimates of human disease burden (communicable and non-communicable; measured as DALYs) are presented in the 2010 GBD study^23^. Twenty-five of the communicable diseases in this work^23^ are caused by pathogens included in our study, allowing us to assess the climate-sensitivity of the highest impact human infections. Nearly three quarters (18/25: 72.0%) had positive evidence of sensitivity to at least one climate driver; sixty-four percent (16/25) had links to primary climate drivers; an additional two (tuberculosis and non-typhoidal salmonellosis) had evidence of links to secondary climate drivers only (Table 3). Considering the GBD study, climate effects were potentially greater and the evidence stronger for bacterial and protozoal diseases than for viruses. The eighteen climate sensitive diseases accounted for 57.8% of total DALY burden (caused by the 25 diseases), or 37.0% if considering primary climate drivers only.

**Table 3:** Disability-Adjusted Life Year estimates (DALYs) for the main pathogens of diseases described the 2010 Global Burden of Disease study^23^ for which the effects of sensitivity to climate drivers have been reviewed.

Disease	All ages DALYs (1000 s) in 2010	DALY impact relative to DALYs for all causes^**a**^	Climate drivers^b^	Median scores for climate drivers	Taxonomic division^c^
Human Immunodeficiency Virus	81547	3.27	—	—	V
Tuberculosis	49396	1.98	Alt	80	B
Rotaviral enteritis	18650	0.75	EWE, M, P, R	20, 20, 20, 20	V
*Escherichia coli*	14436	0.58	Alt, EWE, R, W	80, 40 + 12.5, 80, 80	B
Typhoid and Paratyphoid fever	12239	0.49	M, P, R, T	60, 80, 20, 20	B
Measles	10420	0.42	—	—	V
Syphilis	9578	0.38	—	—	B
Cryptosporidiosis	8372	0.34	EWE, P, R, S, T	80, 60, 60, 60, 80	P
Campylobacter enteritis	7541	0.30	M, R, T	60, 20, 60	B
Shigellosis	7052	0.28	EWE, R	80, 20 + 20	B
Pertussis	7018	0.28	—	—	B
Meningitis (*Neisseria meningitidis*)	5163	0.21	M, P	80, 80	B
*Salmonella enterica* (Non typhoidal)	4847	0.19	P	20	B
Hepatitis B	4674	0.19	R	20	V
Cholera	4463	0.18	CC, EWE, M, O, P, R, S, T, W	5, 80, 22.5, 15, 80, 20, 80, 20, 40	B
Hepatitis A	4351	0.17	EWE, O, R	30, 20, 20	V
Hepatitis E	3715	0.15	EWE, R	20, 20	V
Amoebiasis	2237	0.09	CC, M, R	5, 60, 20	P
Rabies	1462	0.06	Alt, R	20, 50	V
Chlamydia	714	0.03	Alt, EWE, M, R	80, 40, 50, 50	B
Varicella (Chicken pox & Herpes zoster)	581	0.02	M	20	V
Hepatitis C	518	0.02	—	—	V
Trachoma	334	0.01	Alt, EWE	80 + 80, 80	B
Gonorrhea	282	0.01	—	—	B
Trichomoniasis	167	0.01	—	—	P

^a^DALY impact relative to DALYs for all causes (disease DALY/2490385) specified in the 2010 Global Burden of Disease study^23^; ^b^climate drivers with positive median evidence including: altitude (Alt), climate change (CC), extreme weather events (EWE), moisture (M), oscillations (O), particle matter (P), rainfall (R), salinity (S), temperature (T), vegetation (V) and wind (W); median scores for each climate driver. If score includes “ + ” describes median scores for the climate driver for two pathogens; ^c^taxonomic division for the main pathogens causing each disease including: bacteria (B), protozoa (P) and viruses (V).

## Discussion

The methods and results presented within this risk assessment directly address a priority area for research suggested by the World Health Organization, World Organisation for Animal Health (OIE) and European Union, amongst others; namely, contribute to strengthening climate change resilience for infectious diseases, by developing the knowledge base, decision-support tools and data-sets to assess the risk and predict the scale of likely impacts of changes in climate upon infectious pathogens^6, 24–27^. Sensitivity to one or more climate drivers of a pathogen and/or the risk of exposure is a prerequisite for the impact of climate change upon an infectious disease.

A unique aspect of our study is that each step of the process was based on systematically-derived evidence, the results are quantitative as opposed to review work^6^ and the pathogen short-listing methods mean that the most intensively-researched pathogen species have been included in the evaluation. In addition, the process can be repeated to take account of newly published work, and the tools used are open-access and so can be utilized by relevant national and international organisations to help inform policy development on the health impacts of climate change^28^. Research leaders have previously indicated the need for the mounting of ‘a proactive public health response by building an integrated network for environmental and epidemiological data’^16^; the resources developed in this programme of work^18, 20, 21, 29, 30^ contribute substantially to this issue.

This study is the first risk assessment to quantitatively examine the sensitivity to climate of pathogens which have the largest impact upon human and domestic animal health. The results suggest that up to two thirds of high H-index human and domestic animal pathogens which occur in Europe are associated with climate drivers. This result is higher than an estimate from a study examining non-systematically selected human notifiable, haemmorhagic or other emerging European diseases (49%)^17^. Of pathogens with evidence of links to climate drivers, 82% are associated with primary drivers such as temperature, rainfall, humidity and wind speed, which are expected to change with climate change; most climate-sensitive diseases may therefore also respond to climate change.

Whilst having more drivers does not directly indicate that pathogens are more climate sensitive, climate change is expected to impact on many aspects of climate; we therefore assume that being sensitive to a broader range of climate variables means that climate change is more likely to affect a disease or will affect it in more complex ways. Within this study, of those pathogens found to be sensitive to climate, two thirds have more than one climate driver. This indicates that the impact of climate (or climate change) on climate sensitive diseases is multifaceted and complex and cannot be captured by models that consider a single climate driver only. Models describing the impact of climate change on infectious disease burdens should consider all climate drivers known to affect the disease. In particular, moisture and rainfall were identified as the most frequently occurring and most commonly linked drivers. These drivers are often omitted in models of climate change on disease (which frequently focus on temperature change) and, additionally, there remains significant uncertainty in projections of how climate change will affect them in future. Thus, models of the impact of climate change on infectious diseases may be improved as skill in projections for rainfall and moisture increases, and their effect on infectious disease is better quantified and included in models. Commonly occurring combinations of drivers include moisture and temperature, rainfall and temperature, and rainfall and extreme weather events. Models driven by combinations of climate drivers, examining multiple scenarios for changes in them, are urgently needed^31^.

While more than half the pathogens in the prioritized lists affecting humans and domestic animals were different, there were similar numbers with positive evidence of climate drivers. This suggests that these two host types may be equally susceptible to the impact of climate change on infectious disease.

The emergence of new pathogens, or adaptation of those already known, is likely to be governed by a unique combination of events, of which changes in climate are just one. There are many other known drivers of disease emergence. However, climate, and climate change, may impact indirectly on some of these^1^. For example, drought may in future lead to civil unrest or war, and both are associated with breakdown of public health and the spread of certain infectious diseases, such as cholera. Our study has focused on climate drivers (defined as primary or secondary) that impact directly on infectious disease, and does not consider these indirect impacts.

Evidence for pathogen sensitivity to secondary climate drivers should be treated with a degree of caution as some may be a proxy for non-climate drivers such as movement of people or animals, or changes in farming practice, many of which will be associated with climate. For example, the effects of altitude may be driven by environmental temperature or rainfall, but also socio-demographic drivers such as economic or health status, an ability to produce indoor biomass smoke, or outdoor dust exposure.

This study is the first to consider the importance of transmission route on sensitivity to climate and finds that some transmission routes are more sensitive than others. An implication is that vector-borne, foodborne, waterborne and soil-borne pathogens are the most likely to be affected by climate change, while those transmitted by direct contact (sexual or non-sexual) or by fomite are the least likely to be affected by climate change.

This study also suggests for the first time that some taxa are more likely to be affected by climate change. For example bacteria, fungi and viruses mostly had few drivers, (although some bacteria and viruses had many drivers, particularly if vector-borne), compared to protozoa and helminths.

An interesting finding is that pathogens with more climate drivers are found in a larger number of European countries. This relationship could be driven by a larger volume of research undertaken on more widespread pathogens, i.e it is an artifact. Alternatively, it might suggest that widespread pathogens are exposed to a broader range of climates across their range, and therefore that they have broader climate envelopes and are sensitive to a larger number of drivers. For example, spore formation in anthrax facilitates disease outbreaks after both flooding and drought, associating it with both wetter and drier areas.

Zoonotic pathogens were more sensitive to climate than non-zoonotic pathogens suggesting that zoonoses may be particularly likely to be impacted by climate change. As an estimated seventy-five percent of emerging diseases are zoonotic^19^, emerging diseases may therefore be disproportionately sensitive to climate; although here we found that emergence was associated with specific climate drivers, but not the number of climate drivers. Whilst we have not formally investigated the reasons behind the increased sensitivity of zoonotic pathogens, it may be that zoonoses tend to be more researched (by both medical and veterinary research communities), or it may be confounded with transmission route which was associated with both climate sensitivity and being zoonotic. The third possibility, however, is that zoonoses genuinely are more climate sensitive, perhaps because of their wider host and environmental ranges.

The fine-tuning of current surveillance approaches is imperative for early detection of emerging infectious diseases related to climate change, to enhance preparedness and facilitate public health response^17^. This study, which assessed the sensitivity to climate of the most important pathogens of people and domestic animals present in Europe, provides a framework on which to focus the next stage of research, which should look at the nature of associations between pathogens/disease and climate drivers. Importantly, the work used a quantitative approach^21^, so that further climate impact assessments can be made in the future, as new diseases emerge and research identifies new sensitivities to climate. The threat posed by climate change is clearly substantial, with nearly two thirds of pathogens identified as sensitive to changes in at least one climate driver.

Within this study, the H-index was essential for prioritizing large numbers of diseases where alternative metrics were not available. The H-index method has limitations, however, which are acknowledged. These include: the need for manual oversight of search terms to minimize false positives for, for example, pathogens used as model organisms; biases in results because of trends in interest or funding for research in specific pathogens/diseases; underestimation of scientific literature published in non-English languages; effects of citation rates varying with field of research (medical versus veterinary, for example); effects of different bibliographic databases containing different literature sources; and H-index results are susceptible to a lag in time-to-publication, with newly emerging pathogens, in particular, likely to be under-represented^32^.

This study demonstrates that some of the most important diseases present in Europe, in terms of human mortality and morbidity (recorded as DALYs in the 2010 GBD study^23^), are sensitive to climate and may therefore be affected by climate change. Nearly three quarters of diseases in the 2010 GBD study^23^ that were assessed, had evidence of at least one climate driver, while these diseases accounted for a smaller percentage (58%) of the total DALYs. This reduction arises from the highest DALY disease, HIV, not being found to be sensitive to climate. Interestingly, the disease with the second highest DALY, TB, was found to be associated with a single secondary driver, altitude, which could be describing the effect of other socio-demographic factors. This clearly illustrates the difficulty of trying to dissociate climate from other drivers, particularly those of anthropogenic origin. Nevertheless, pathogens with primary climate drivers accounted for nearly two fifths of the total DALYs, indicating that climate change may impact significantly on human health and well-being in Europe. Important diseases are not, therefore, resilient to the effects of climate change.

## Methods

To estimate pathogen impact for a large number of pathogens, we used the H-index methodology to obtain H-indices for 3,628 human and animal pathogen species, and then selected for detailed assessment the top one hundred human and top one hundred domestic animal pathogens which occur in Europe, and which cause significant clinical disease. As some pathogens appeared within both sets (for more information see^21^), this gave a total of 157 pathogens (see Table S1 for human pathogens, Table S2 for domestic animal pathogens, Table S3 for domestic hosts and Table S4 for European countries). Published scientific literature was systematically searched for the co-occurrence of pathogen names (and synonyms) and a defined list of climate drivers (the Supplementary Information includes the search protocol and Table S5 describes the climate terms). This literature was systematically reviewed, seeking evidence of links suggesting sensitivity of a pathogen to climate drivers, taking account of the strength of evidence and the transmission routes of the infectious agent (see Supplementary Information for further detail). We consider two types of climate driver: primary, those based on climate variables i.e. moisture, temperature, rainfall, windspeed, and secondary, which are combinations, consequences or proxies of these i.e. altitude, vegetation and salinity. These drivers can act directly, in that they have a consequence on the disease, or indirectly, in that they have an impact on another driver, which has a consequence on the disease. We considered the climate drivers of pathogens classed as zoonotic versus not zoonotic, and emerging versus non-emerging (defined in the Supplementary Information). Finally, we apply our methodology to the highest impact human diseases included within the 2010 Global Burden of Disease study^23^, to quantify the proportion of DALYs that arise from climate sensitive diseases.

### Data Availability

The datasets generated or analysed during this study are included in this published article (and its Supplementary Information file) or publically available at https://eid2.liverpool.ac.uk/.

## Electronic supplementary material

Supplementary_Information
