# Author Correction: Systematic Assessment of the Climate Sensitivity of Important Human and Domestic Animals Pathogens in Europe

**DOI:** 10.1038/s41598-018-24606-6

**Published:** 2018-04-25

**Authors:** K. Marie McIntyre, Christian Setzkorn, Philip J. Hepworth, Serge Morand, Andrew P. Morse, Matthew Baylis

**Affiliations:** 1 Department of Epidemiology and Population Health, Institute of Infection and Global Health, University of Liverpool, Leahurst Campus, Neston, Cheshire CH64 7TE UK; 2 NIHR Health Protection Research Unit in Emerging and Zoonotic Infections, Liverpool, L69 7BE UK; 3 CNRS ISEM - CIRAD ASTRE, Faculty of Veterinary Technology, Kasetsart University, Bangkok, Thailand; 4 Department of Helminthology, Faculty of Tropical Medicine, Mahidol University, Bangkok, 10400 Thailand; 5 Department of Geography and Planning, School of Environmental Sciences, Roxby Building, University of Liverpool, Liverpool, L69 7ZT UK

Correction to: *Scientific Reports* 10.1038/s41598-017-06948-9, published online 02 August 2017

This Article contains an error in the legend of Figure 1.

“(b) Proportion of primary (in dark grey) compared to secondary (in light grey) drivers associated with different numbers of climate drivers”

should read:

“(b) Proportion of primary (in light grey) compared to secondary (in dark grey) drivers associated with different numbers of climate drivers”

Additionally, in the Results section,

“By contrast, the distributions for protozoa (N = 7) and helminths (N = 6) were left-skewed or bell-shaped (Fig. 2c,d, respectively).”

should read:

“By contrast, the distributions for protozoa (N = 7) and helminths (N = 6) were left-skewed or bell-shaped (Fig. 2d,c, respectively).”

