# Supplementary material for: Systematic Assessment of the Climate Sensitivity of Important Human and Domestic Animals Pathogens in Europe
Source: Sci Rep. 2017 Aug 2;7:7134. doi: 10.1038/s41598-017-06948-9 (PMC5541049; doi:10.1038/s41598-017-06948-9)
Supplement: Supplementary file 1 — Supplementary_Information [file 41598_2017_6948_MOESM1_ESM.pdf]

Supplementary Information for

Systematic Assessment of the Climate Sensitivity of Important Human and Domestic Animals Pathogens in Europe

Running title

Infectious Disease Climate Sensitivity

Authors K. Marie McIntyre, Christian Setzkorn, Philip J. Hepworth, Serge Morand, Andrew P. Morse and Matthew Baylis

Further methods

H-index methodology

We previously reported the use of the quantitative H-(Hirsch-)index methodology [1] for pathogens/diseases. This metric gauges the volume and citation rate of research on diseases or pathogens, serving as a proxy for more comprehensive measures including Disability-Adjusted Life Year estimates (DALYs) [1]. A tool like the H-index is essential for prioritizing large numbers of diseases, as lack of data would prohibit the use of more direct measures for the majority of infections. Furthermore, the H-index can also be readily applied to infectious diseases of animals [2], while alternative methods for the prioritization of animal diseases would generally use different criteria to those used to prioritize human infections (such as economic impact).

Pathogen Information

Information on the pathogens of humans and domestic animals, including the number of European countries where there is evidence for their presence was taken from the ENHanCEd Infectious Diseases 2 (EID2) database [3,4]. This database integrates published data sources (NCBI nucleotide metadata, and PubMed abstracts) allowing conclusions about pathogens, hosts and vectors to be drawn using evidence from individual reports, for example, that a certain pathogen infects a certain host, and was present in a certain country at a certain time. For our analysis, domestic animals were defined as mammals and birds farmed or regularly kept as pets in Europe (see Table S3). Europe included European states and transcontinental states (see Table S4).

Climate driver literature searching protocol

Systematic literature searches were used to minimize bias in the identification of papers which possibly describe the climate drivers of pathogens. We did not publish a protocol in advance of the review.

Information sources - Searches of published scientific literature were undertaken in December 2011 using PubMed [5].

Eligibility criteria - Searches were restricted to the years 1948 to 2011, inclusive, and there was no geographical restriction on where the papers originated from. English is used in PubMed, however searches also include foreign-language publication title translations. All literature in the PubMed database has been published.

Searches - Searches were undertaken using search phrases specified in quotation marks (“”), the ‘topic’ search field and with no lemmatization. A list of climate driver terms

(n=190, see Table S5) was compiled by the study authors using manual searches of scientific literature to identify keywords or phrases describing climate drivers likely to impact pathogens. Search phrases were compiled which included a climate driver term, the pathogen scientific name, alternative names, synonyms and alternative spellings according to NCBI Taxonomy [6]. Virus searches also included synonyms and acronyms from the NCBI Taxonomy database[4] and International Committee on Taxonomy of Viruses [6,7], and the term ‘virus’, and excluded other entities (viral or non-viral) which shared acronyms; the search terms describing pathogens are available in the EID2 [3,4]. The Boolean operator ‘AND’ was used to link each climate driver term with the pathogen name phrase, ‘OR’ was used between each element of the pathogen name, and ‘NOT’ was used where necessary to exclude viral or non-viral entities sharing virus acronyms. Individual literature searches were undertaken for combinations of every pathogen and climate term.

Systematic reviewing of literature

The individual searches on each climate term and pathogen combination produced a database of approximately 27,000 rows of data, each linked to a published paper. The papers described within the database were stratified according to pathogen name and a climate driver grouping term, hereafter referred to as climate drivers, (e.g. the climate terms ‘ambient temperature’ and ‘air temperature’ were included in a grouping term ‘temperature’; see Table S5), and the results were pooled for all of the terms per group. Each set of pooled papers was then prioritized for reading, according to the impact factor

of the journal in which they were published and the year of publication (with the highest impact factor and most recent publication given highest priority).

The reviewing and scoring procedure for each set of papers is summarized (see Figure S1). First, papers were accepted or rejected from the study based on two inclusion criteria: (i) whether the paper was about a climate driver of disease (for example, whether temperature refers to the climate variable rather than fever), and (ii) whether the paper investigated a quantitative relationship between the level of the driver and disease (i.e. quantified the health outcome, if not, it is not possible to draw conclusions about how a change in the driver might affect disease). The second step was to score accepted papers based upon a number of data categories: the type of analysis; the strength of statistical evidence for an effect of a climate driver acting upon a pathogen; and whether the publication suggested that the climate driver effect did or did not affect the pathogen. The acceptance or rejection of papers and the scoring of accepted papers was undertaken by one of two researchers, using a standardized data collection form. Prior to the reviewing process, a subset of studies (N=100) were accepted or rejected and scored by both researchers, and any differences were resolved by discussion leading to agreement on the interpretation of data categories. The final scores were combined multiplicatively. (Outcomes ranged from 80 to -80. A score of 80 corresponded to the strongest evidence for an effect of climate on disease, and a score of -80 corresponded to the strongest evidence for the lack of an effect of climate on disease). In each case, a maximum of 20 papers were examined to look for the effect of a climate driver upon a pathogen, and a maximum of 5 papers were scored. This minimized biases arising as a result of the

number of publications describing a pathogen/climate driver combination. The reviewing procedure, where papers were accepted or rejected for further systematic reviewing was undertaken for studies describing N=4959 pathogen and climate driver relationships. Of these, N=661 relationships were accepted within the review and scored for evidence describing a quantitative relationship between the level of the climate driver and disease (see Figure S1), from a total of N=542 scientific publications.

Transmission routes for pathogens

We obtained the major transmission routes for our 157 pathogens using two methods. First, we undertook manual searches of text books on the infectious diseases of humans and relevant domestic animals [8-15]. These results were supplemented with output from semi-automated literature searches which recorded the frequency of papers associated with combinations of pathogens and certain keywords/phrases that describe potential transmission routes; transmission routes were confirmed by reading the papers identified for each pathogen/keyword combination. Transmission routes were not considered for pathogens which are part of the natural biome.

Emerging/zoonotic pathogen status

Information on whether pathogens were zoonotic, non-zoonotic, emerging and not emerging was examined based upon previously published information [16,17]. If not included in earlier work or if their status had changed due to more recent scientific evidence, updated pathogen information was based upon the same definitions. Zoonotic pathogens were classified as those naturally transmitted between vertebrate (non-human)

animals and humans, not including species which have recently evolved from animal pathogens but are no longer transmitted between animals and humans [16,18]. Emerging pathogens are those that have appeared in a host population for the first time (including newly-evolved strains), or have occurred previously but are increasing in incidence or expanding into areas where they had not previously been reported [16,18]. Pathogens needed to have emerged in several geographically distinct areas to be 'emerging'.

Statistical analyses

The relationship between the frequency of climate drivers and transmission routes associated with pathogens was explored using Pearson's product-moment correlation analyses. The relationship between the number of European countries with evidence of pathogen presence and the frequency of climate drivers was examined using a linear model which also included the frequency of transmission routes associated with a pathogen as a covariate. Pearson's Chi-squared tests with Yates' continuity correction were used to establish if pathogens with positive evidence of climate drivers were more or less likely to be zoonotic compared to affecting humans or animals-only, or classed as emerging compared to not emerging. Binomial logistic regression models were used to explore relationships between climate drivers and emerging pathogen status in models which also explored the effects of zoonotic disease status, (quartiles describing) H-indices for pathogens, and taxonomic division. Binomial logistic regression models were also used to explore relationships between evidence for the number of and certain transmission routes and zoonotic status, including (quartiles describing) H-indices for pathogens. After climate drivers or transmission routes with some evidence of an effect

were identified (P<0.3), these were then incorporated into multivariable models using a forward stepwise approach. R was used for all statistical analyses [19], with statistical significance determined by a P-value of 0.05 or less.

Figure S1. Reviewing process for each paper and climate driver and pathogen combination.

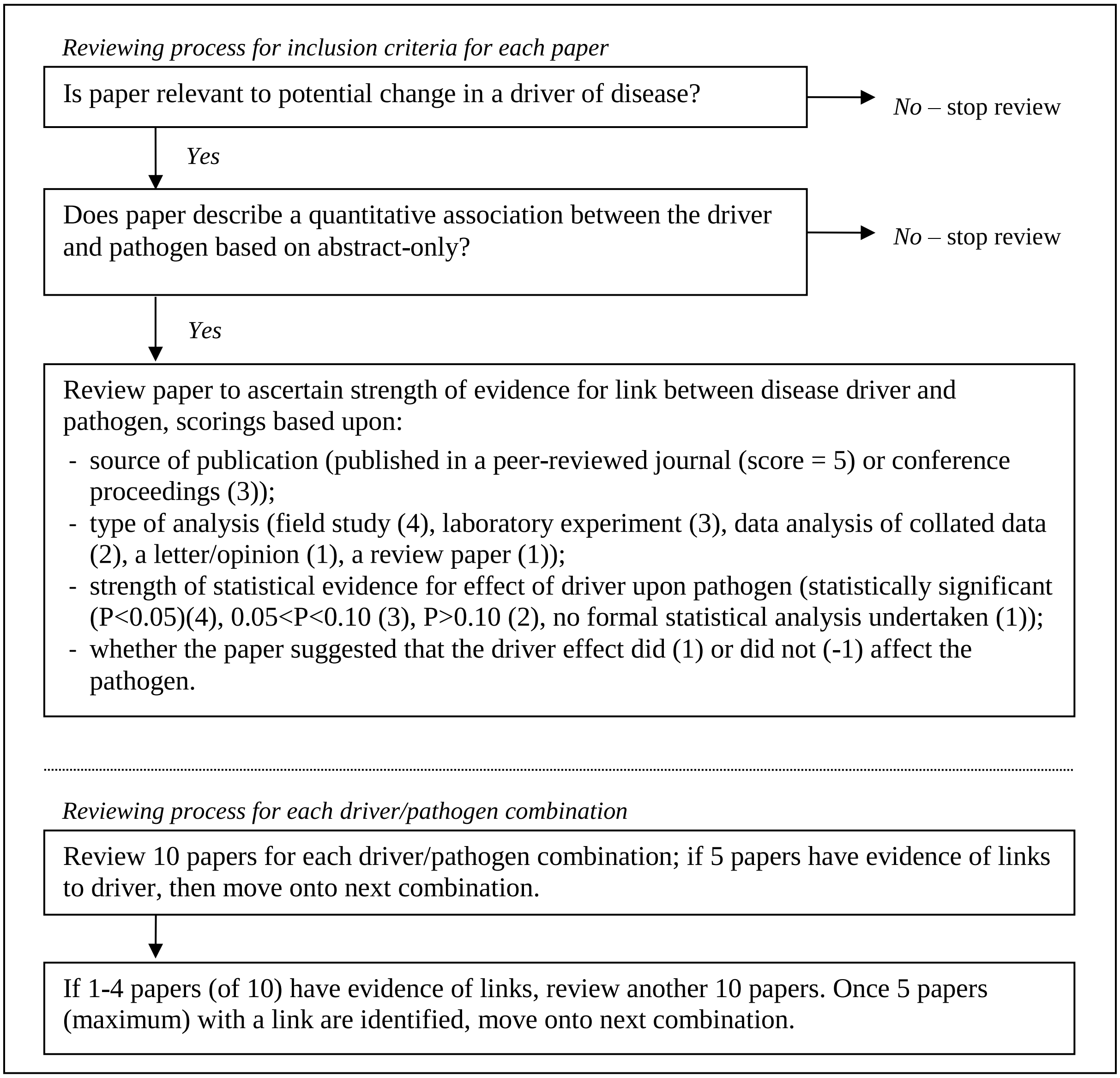

Table S1. Top 100 human pathogens of Europe, prioritized according to the H-index methodology [1]. **[a]** Pathogens include those which are zoonotic (Z), non-zoonotic (NZ), emerging (E) and not emerging (NE) according either to [16,17] or given a new status (NS) in this work. Pathogens also included in the list of top 100 animal pathogens are noted (A). [b] Climate drivers with positive evidence include: altitude (Alt), climate change (CC), extreme weather events (EWE), moisture (M), oscillations (O), particle matter (P), rainfall (R), salinity (S), temperature (T), vegetation (V) and wind (W). [c] Transmission routes include: airborne (via inhalation) (air), direct (non-sexual) contact (dcont), fomite (fom), foodborne (via ingestion) (food), direct sexual contact - (sex), soil-borne (soil), vector-borne (vect) and waterborne (wat). [d] Taxonomic divisions (TD) of pathogens include: bacteria (B), fungi (F), helminthes (H), protozoa (P) and viruses (V).

Pathogen name[a]	Climate drivers[b]	Transmission routes[c]	TD[d]	H-index score	Pathogen name[a]	Climate drivers[b]	Transmission routes[c]	TD[d]	H-index score
Escherichia coli, Z, E, A	Alt, EWE, W	dcont, wat, food	B	524	*Streptococcus pyogenes*, Z, E		dcont, wat, air, food	B	113
Human Immunodeficiency Virus 1, NZ, E		sex	V	410	*Bacillus cereus*, Z, NE, A	P	air, food	B	111
Human Immunodeficiency Virus 2, NZ, E		sex	V	399	*Aspergillus niger*, Z, NE	Alt, M, S, W	air	F	110
Hepatitis C Virus, NZ, E		sex	V	289	*Burkholderia cepacia*, NZ, NE		dcont, soil, wat, air, fom	B	107
Staphylococcus aureus, Z, E, A	EWE, M, S	dcont, fom, food	B	271	*Clostridium botulinum*, Z, E, A	P, W	soil, food	B	106
Human Herpesvirus 4, NZ, NE		dcont	V	257	Encephalomyocarditis Virus, Z, NE, A	M	wat, fom, food	V	105
Helicobacter pylori, Z, NE, A	Alt, R	dcont, sex, fom, food	B	246	*Yersinia pestis*, Z, E, A	CC, O, R	dcont, vect, food	B	105
Hepatitis B Virus, NZ, E	R	dcont, sex	V	246	*Streptococcus mutans*, NZ, NE			B	104
Pseudomonas aeruginosa, Z, E, A	M	wat, air, fom, food	B	243	*Aggregatibacter actinomycetemcomitans*, Z, NE		dcont	B	101
Mycobacterium tuberculosis, Z, E	Alt	air	B	238	*Clostridium perfringens*, Z, NE, A	EWE, P	soil, food	B	101
Human Papillomavirus, NZ, E		dcont, sex	V	235	*Serratia marcescens*, Z, E	T	dcont, fom	B	100
Bacillus subtilis, Z, NE	M	food	B	219	*Yersinia pseudotuberculosis*, Z, NE, A	EWE, R	dcont, wat, food	B	99
Listeria monocytogenes, Z, E, A	EWE, P, R, S, T	food	B	207	*Entamoeba histolytica*, Z, NE, A	CC, M, R	wat, food	P	98

Streptococcus pneumoniae, Z, E	CC, M, R, T, W	dcont	B	199	*Leishmania donovani*, Z, E, A	Alt, M	vect	P	98
Candida albicans, Z, E, A		dcont, sex, fom	F	181	*Bacteroides fragilis*, Z, NE, A	T		B	97
Human Herpesvirus 1, NZ, E		dcont, sex	V	171	*Gibberella moniliformis*, NZ, E	M, R, T	food	F	97
Respiratory Syncytial Virus, NZ, NE	Alt, M, R, W	air, fom	V	164	West Nile Virus, Z, E, A	CC, EWE, M, R, T, V	vect	V	97
Human Herpesvirus 5, NZ, E		dcont, sex	V	159	Human Herpesvirus 2, NZ, E		dcont, sex	V	96
Haemophilus influenzae, NZ, E		dcont, air	B	148	Rabies Virus, Z, E, A	Alt, R	dcont	V	96
Lymphocytic Choriomeningitis Virus, Z, NE, A		dcont, air	V	148	Hepatitis A Virus, Z, E	EWE, O, R	dcont, sex, wat, food	V	95
Toxoplasma gondii, Z, E, A	Alt, CC, EWE, M, R	wat, food	P	148	Human Herpesvirus 6, NZ, NE		dcont	V	94
Klebsiella pneumoniae, Z, E, A	M	dcont, air	B	146	*Fusarium oxysporum*, NZ, E	P	air, food	F	93
Vibrio cholerae, NZ, E	CC, EWE, M, O, P, R, S, T, W	wat, food	B	145	*Mycoplasma pneumoniae*, NZ, NE	M, T	air	B	93
Borrelia burgdorferi, Z, E, A	Alt, CC, M, O, R, T, V	vect	B	144	*Cryptosporidium parvum*, Z, E, A	EWE, P, R, S, T	dcont, wat, food	P	92
Chlamydophila pneumoniae, Z, NE, A	M	air	B	143	*Enterobacter cloacae*, Z, NE, A	P	dcont, food	B	90
Shigella flexneri, Z, NE	EWE, R	dcont, sex, wat, vect, food	B	142	*Aeromonas hydrophila*, Z, E, A	EWE, M, R, S	wat, food	B	89
Human Herpesvirus 8, NZ, E		dcont, sex	V	140	*Acinetobacter baumannii*, NZ, NE, A	M, P, T	soil, wat, air, fom, food	B	88
Escherichia coli O157:h7, Z, E	EWE, R	dcont, wat, food	B	138	*Candida glabrata*, Z, E		dcont, sex, fom	F	87
Human T-lymphotropic Virus 1, NZ, E	Alt	dcont, sex	V	137	*Moraxella catarrhalis*, NZ, NE	R	air	B	87
Neisseria gonorrhoeae, NZ, E		sex	B	136	*Salmonella enterica* subsp. *enterica* serovar enteritidis, Z, E, A	P	dcont, food	B	87
Influenza A Virus, Z, E, A	CC, M, P, R, S, T	air	V	135	*Treponema pallidum*, NZ, E		dcont, sex, food	B	87
Legionella pneumophila, NZ, E	P, R, W	air	B	133	*Trichomonas vaginalis*, NZ, E		sex	P	87
Enterococcus faecalis, Z, E	S	dcont	B	132	*Rhizopus oryzae*, Z, NE		air	F	86
Mycobacterium bovis, Z, E, A	Alt, EWE	air, food	B	132	Hepatitis E Virus, Z, E	EWE, R	wat, food	V	83
Campylobacter jejuni, Z, E, A	M, R	dcont, wat, food	B	130	Human Parvovirus b19, NZ, E		dcont, air	V	81
Neisseria meningitidis, NZ, E	M, P	dcont, air	B	130	*Proteus mirabilis*, Z, NE, A			B	80
Chlamydia trachomatis, Z, E	Alt, EWE, M, R	dcont, sex	B	129	*Shigella dysenteriae*, Z, E	R	dcont, wat, vect, food	B	80
Clostridium difficile, Z, E, A	R	dcont, fom, food	B	127	*Stenotrophomonas maltophilia*, NZ, NE		wat, fom, food	B	80
Cryptococcus neoformans, Z, E, A	M, R	air	F	126	*Bacillus licheniformis*, Z, NE, A	P	food	B	78

Yersinia enterocolitica, Z, E, A		dcont, food	B	126	*Mycoplasma genitalium*, NZ, NE		sex	B	78
Mouse Mammary Tumour Virus, Z, E, NS			V	125	*Trichinella spiralis*, Z, E, A	Alt, M	food	H	78
Mycobacterium avium, Z, E	Alt, P, R	wat, air, food	B	125	*Bartonella henselae*, Z, NE, A	M, R	dcont, vect	B	77
Bacillus anthracis, Z, E, A	CC, M, P, R, T, V, W	soil, air, fom, food	B	122	*Salmonella enterica* subsp. *enterica* serovar typhimurium, Z, E, A	M, P, R, T	wat, food	B	77
Bordetella pertussis, NZ, E		air	B	122	*Brucella abortus*, Z, E, A	M, O	dcont, air, food	B	76
Measles Virus, Z, E		air	V	119	*Candida tropicalis*, Z, NE		dcont, sex, fom	F	76
Human Enterovirus C, NZ, NE	M, S	dcont, air, fom	V	118	*Pseudomonas stutzeri*, NZ, NE			B	76
Enterococcus faecium, Z, E	T	dcont	B	116	SARS coronavirus, Z, E, NS	T	dcont, air, fom	V	76
Staphylococcus epidermidis, Z, E, A	P	fom, food	B	114	*Enterobacter aerogenes*, Z, NE, A	T	dcont, food	B	75
Human Herpesvirus 3, NZ, E	M	dcont, air	V	113	*Francisella tularensis*, Z, E, A	R, T	wat, air, vect, food	B	74
Porphyromonas gingivalis, Z, NE	T		B	113	*Vibrio parahaemolyticus*, Z, E	EWE, O, P, R, S, T	food	B	74

Table S2. Top 100 domestic animal pathogens of Europe prioritized according to the H-index methodology [1]. [a] The emerging and zoonotic definitions are the same as for Table S1. Pathogens also included in the list of top 100 human pathogens are noted (H). [b] Climate drivers with positive evidence include: altitude (Alt), climate change (CC), extreme weather events (EWE), moisture (M), oscillations (O), particle matter (P), rainfall (R), salinity (S), temperature (T), vegetation (V) and wind (W). [c] Transmission routes include: airborne (via inhalation) (air), direct (non-sexual) contact (dcont), fomite (fom), foodborne (via ingestion) (food), direct sexual contact - (sex), soil-borne (soil), vector-borne (vect) and waterborne (wat). [d] Taxonomic divisions (TD) of pathogens include: bacteria (B), fungi (F), helminthes (H), protozoa (P) and viruses (V).

Pathogen name[a]	Climate drivers[b]	Transmission routes[c]	TD[d]	H-index score	Pathogen name[a]	Climate drivers[b]	Transmission routes[c]	TD[d]	H-index score
Escherichia coli, Z, E, H	Alt, EWE, W	dcont, wat, food	B	524	*Salmonella enterica* subsp. *enterica* serovar typhimurium, Z, E, H	M, P, R, T	wat, food	B	77
Staphylococcus aureus, Z, E, H	EWE, M, S	dcont, fom, food	B	271	*Brucella abortus*, Z, E, H	M, O	dcont, air, food	B	76
Helicobacter pylori, Z, NE, H	Alt, R	dcont, sex, fom, food	B	246	*Enterobacter aerogenes*, Z, NE, H	T	dcont, food	B	75
Pseudomonas aeruginosa, Z, E, H	M	wat, air, fom, food	B	243	*Francisella tularensis*, Z, E, H	R, T	wat, air, vect, food	B	74
Listeria monocytogenes, Z, E, H	EWE, P, R, S, T	food	B	207	*Haemonchus contortus*, NZ, NE, NS	CC, EWE, M, R, T	food	H	74
Murine Leukemia Virus, Z, E, NS			V	184	*Neospora caninum*, NZ, NE, NS	M, R, T	wat, food	P	72
Candida albicans, Z, E, H		dcont, sex, fom	F	181	Cowpox Virus, Z, E, NS		dcont	V	71
Lymphocytic Choriomeningitis Virus, Z, NE, H		dcont, air	V	148	Bovine Herpesvirus 1, NZ, NE, NS	Alt, T	dcont, sex, air	V	70
Toxoplasma gondii, Z, E, H	Alt, CC, EWE, M, R	wat, food	P	148	*Citrobacter freundii*, NZ, E, NS		food	B	70
Klebsiella pneumoniae, Z, E, H	M	dcont, air	B	146	Feline Leukemia Virus, NZ, NE, NS		dcont	V	69
Borrelia burgdorferi, Z, E, H	Alt, CC, M, O, R, T, V	vect	B	144	*Fasciola hepatica*, Z, E, NS	Alt, CC, EWE, M, R, T, V	vect, food	H	68
Chlamydophila pneumoniae, Z, NE, H	M	air	B	143	Reticuloendotheliosis virus, NZ, NE, NS		dcont	V	68
Influenza A Virus, Z, E, H	CC, M, P, R, S, T	air	V	135	*Coxiella burnetii*, Z, E, NS	M, P, R, W	air, vect, food	B	67
Mycobacterium bovis, Z, E, H	Alt, EWE	air, food	B	132	*Mannheimia haemolytica*, Z, NE, NS	M, P, T	air	B	67

Campylobacter jejuni, Z, E, H	M, R	dcont, wat, food	B	130	Infectious Bronchitis Virus, NZ, NE, NS		air	V	66
Clostridium difficile, Z, E, H	R	dcont, fom, food	B	127	*Klebsiella oxytoca*, NZ, NE, NS	P	fom, food	B	66
Cryptococcus neoformans, Z, E, H	M, R	air	F	126	*Ascaris suum*, Z, NE, NS	Alt	food	H	65
Yersinia enterocolitica, Z, E, H		dcont, food	B	126	Borna Disease Virus, Z, E, NS		dcont, fom, food	V	65
Bacillus anthracis, Z, E, H	CC, M, P, R, T, V, W	soil, air, fom, food	B	122	Bovine Leukemia Virus, NZ, NE, NS		dcont, vect	V	65
Staphylococcus epidermidis, Z, E, H	P	fom, food	B	114	*Campylobacter coli*, Z, NE, NS	T	dcont, wat, food	B	65
Bacillus cereus, Z, NE, H	P	air, food	B	111	Canine parvovirus, NZ, NE, NS		dcont, fom, food	V	65
Clostridium botulinum, Z, E, H	P, W	soil, food	B	106	Parainfluenza Virus 5, Z, NE, NS			V	65
Suid Herpesvirus 1, NZ, NE, NS	M, W	dcont, sex, air, food	V	106	*Pasteurella multocida*, Z, NE, NS	M, P	dcont, wat, air	B	65
Encephalomyocarditis Virus, Z, NE, H	M	wat, fom, food	V	105	Porcine Circovirus, NZ, E, NS	P	dcont	V	65
Yersinia pestis, Z, E, H	CC, O, R	dcont, vect, food	B	105	Porcine Reproductive and Respiratory Syndrome Virus, NZ, E, NS	M, T	dcont, sex, air	V	65
Bovine Spongiform Encephalopathy Agent, Z, E, NS		food	V	101	Bluetongue Virus, NZ, E, NS	Alt, CC, M, T, V, W	vect	V	64
Clostridium perfringens, Z, NE, H	EWE, P	soil, food	B	101	*Chlamydophila abortus*, Z, E, NS	P	dcont, air, food	B	63
Yersinia pseudotuberculosis, Z, NE, H	EWE, R	dcont, wat, food	B	99	*Chlamydophila psittaci*, Z, E, NS	P	dcont, air, fom	B	63
Entamoeba histolytica, Z, NE, H	CC, M, R	wat, food	P	98	*Enterococcus hirae*, Z, NE, NS			B	63
Leishmania donovani, Z, E, H	Alt, M	vect	P	98	Gallid Herpesvirus 2, NZ, NE, NS	P	air, food	V	63
Bacteroides fragilis, Z, NE, H	T		B	97	*Anaplasma phagocytophilum*, Z, E, NS	Alt, CC, M, R, T, V	vect	B	62
West Nile Virus, Z, E, H	CC, EWE, M, R, T, V	vect	V	97	Equine Infectious Anemia Virus, NZ, NE, NS		vect	V	62
Rabies Virus, Z, E, H	Alt, R	dcont	V	96	*Streptococcus agalactiae*, Z, E, NS		sex	B	62
Bovine Papillomavirus, NZ, E, NS		dcont	V	95	*Echinococcus granulosus*, Z, E, NS	CC, EWE, M, R, T	food	H	61
Newcastle Disease Virus, Z, NE, NS	P	dcont, air, fom, food	V	93	Equine Arteritis Virus, NZ, E, NS		sex, air, fom	V	61
Cryptosporidium parvum, Z, E, H	EWE, P, R, S, T	dcont, wat, food	P	92	Maedi-Visna Virus, NZ, NE, NS		dcont, air	V	61
Enterobacter cloacae, Z, NE, H	P	dcont, food	B	90	Canine Distemper Virus, NZ, E, NS	R	dcont, air	V	60
Aeromonas hydrophila, Z, E, H	EWE, M, R, S	wat, food	B	89	Chicken Anaemia Virus, NZ, E, NS		dcont, air, food	V	60

Foot-and-Mouth Disease Virus, Z, NE	M, W	dcont, air, fom	V	89	Equid Herpesvirus 1, NZ, E, NS		dcont, air, fom	V	60
Acinetobacter baumannii, NZ, NE, H	M, P, T	soil, wat, air, fom, food	B	88	Infectious Bursal Disease Virus, NZ, NE, NS		wat, fom, food	V	60
Salmonella enterica subsp. *enterica* serovar enteritidis, Z, E, H	P	dcont, food	B	87	Transmissible Gastroenteritis Virus, NZ, NE, NS	M, T	dcont, fom, food	V	60
Classical Swine Fever Virus, NZ, NE, NS	M, T, W	dcont, air, fom, vect	V	84	*Actinobacillus pleuropneumoniae*, NZ, E, NS		dcont, air, fom	B	59
Bovine Viral Diarrhoea Virus 1, NZ, E, NS	Alt	dcont, sex, fom, vect	V	80	Feline Calicivirus, Z, E, NS		dcont, fom	V	59
Feline Immunodeficiency Virus, NZ, E, NS		dcont	V	80	Myxoma Virus, NZ, NE, NS	W	dcont, air, vect	V	59
Proteus mirabilis, Z, NE, H			B	80	*Encephalitozoon cuniculi*, Z, E, NS	S	dcont, wat, food	F	58
Mycobacterium avium subsp. *paratuberculosis*, NZ, E, NS	P, R	dcont, fom, food	B	79	Rotavirus A, Z, E, NS	EWE, M, P, R	dcont, air	V	58
Scrapie Agent, NZ, NE, NS		dcont, fom, food	V	79	*Campylobacter fetus*, Z, E, NS		dcont, food	B	57
Bacillus licheniformis, Z, NE, H	P	food	B	78	Fowlpox Virus, NZ, NE, NS		air, fom, vect	V	57
Trichinella spiralis, Z, E, H	Alt, M	food	H	78	*Leishmania infantum*, Z, E, NS	Alt, M, R, T, W	vect	P	57
Bartonella henselae, Z, NE, H	M, R	dcont, vect	B	77	*Trichostrongylus colubriformis*, Z, NE, NS	M, R	wat, food	H	57

Table S3. Animal species including humans for which pathogens have been studied, including domestic animals we eat or companion animals we keep as pets, and exotic animals also used as food sources or as pets.

Scientific name	Common name	Scientific name	Common name
Agapornis personata	Masked lovebird	*Lama glama*	Lama
Agapornis roseicollis	Rosy-faced lovebird	*Lama pacos*	Alpaca
Anas platyrhynchos	Domestic duck	*Meleagris gallopavo*	Turkey
Anser anser	Domestic goose	*Melopsittacus undulatus*	Budgerigar
Bison bison	American bison	*Meriones unguiculatus*	Mongolian gerbil
Bison bonasus	European bison	*Mesocricetus auratus*	Syrian golden hamster
Bos indicus	Zebu	*Mus musculus*	House mouse
Bos Taurus	Cow	*Mustela putorius furo*	Domestic ferret
Camelus dromedarius	Dromedary	*Numida meleagris*	Helmeted guineafowl
Canis lupus familiaris	Domestic dog	*Nymphicus hollandicus*	Cockatiel
Capra hircus	Domestic goat	*Oryctolagus cuniculus*	Domestic rabbit
Capreolus capreolus	Roe deer	*Ovis aries*	Sheep
Cavia porcellus	Domestic guinea pig	*Ovis aries musimon*	Mouflon
Cervus elaphus	Red deer	*Pavo cristatus*	Blue peafowl
Chinchilla lanigera	Chinchilla	*Phasianus colchicus*	Ring-necked pheasant
Columba livia	Domestic pigeon	*Rangifer tarandus*	Reindeer
Cricetus cricetus	Common hamster	*Rattus norvegicus*	Brown rat
Dama dama	Fallow deer	*Rattus rattus*	Black rat
Equus asinus	Domestic donkey	*Rhombomys opimus*	Great Gerbil
Equus caballus	Domestic horse	*Serinus canaria*	Canary
Felis catus	Domestic cat	*Struthio camelus*	Ostrich
Gallus gallus	Chicken	*Sus scrofa*	Wild boar
Homo sapiens	Humans	*Sus scrofa domesticus*	Domestic pig
Lagopus lagopus scotica	Red grouse		

Table S4. European states and transcontinental states included in the study, within which information on pathogens was searched using the ENHanCEd Infectious Diseases 2 (EID2) database [3,4].

Albania	Gibraltar	Norway
Andorra	Greece	Poland
Armenia	Hungary	Portugal
Austria	Iceland	Republic of Belarus
Belgium	Ireland	Romania
Bosnia-Herzegovina	Italy	San Marino
Bulgaria	Kosovo	Scotland
Channel Islands	Latvia	Serbia
Croatia	Liechtenstein	Sicily
Cyprus	Lithuania	Slovakia
Czech Republic	Luxembourg	Slovenia
Denmark	Macedonia (Republic)	Spain
England	Malta	Sweden
Estonia	Moldova	Switzerland
Finland	Monaco	Ukraine
France	Montenegro	Vatican City
Georgia (Republic)	Netherlands	Wales
Germany	Northern Ireland	

Table S5. Climate terms grouped into eleven sets (hereafter referred to as climate drivers) for which paper scoring was undertaken (see Figure S1).

Climate driver grouping	Climate driver	Climate driver grouping	Climate driver
**Altitude**	Altitude	**Salinity**	Salinity
**Climate change**	Climate change	**Temperature**	Air temperature
**Extreme weather event**	Cyclone		Air temperatures
	Extreme weather		Ambient temperature
	Flood		Ambient temperatures
	Flooding		Elevated temperature
	Heat wave		Elevated temperatures
	Hurricane		Freezing
	Storm		High temperature
**Moisture**	Absolute humidity		High temperatures
	Dew point		Higher temperature
	Humid		Higher temperatures
	Humidity		Increased temperature
	Relative humidity		Increased temperatures
	Saturation deficit		Low temperature
	Soil moisture		Low temperatures
**Oscillations**	El Niño		Lower temperatures
	La Niña		Maximum temperature
	North Atlantic Oscillation		Mean annual temperature
	Pacific Decadal Oscillation		Mean temperature
**Particle matter**	Dust		Minimum temperature
	Dusty		Summer temperatures
	Turbidity		Warm temperature
**Rainfall**	Annual rainfall		warm temperatures
	Drought		Warm years
	Dry		Warmer temperatures
	Heavy rain		Winter temperatures
	High rainfall	**Wind**	Wind
	Monsoon		Wind speed
	Precipitation		Windy
	Rain	**Vegetation**	NDVI
	Rainfall		
	Rainy season		
	Runoff		
	Wet conditions		
	Wet season		

Table S6. The results of a systematic review of the scientific literature examining the sensitivity to climate drivers of the top 100 human and domestic animal pathogens of Europe [2]. For each paper reviewed, final scores were based upon the type of analysis, the strength of statistical evidence for an effect of a climate driver acting upon a pathogen and whether the publication suggested that the climate driver effect did or did not affect the pathogen. For each pathogen and climate driver combination with a positive median score, the final minimum, median and maximum scores from papers examined are presented, including information on the PMIDs (also indicating the number of papers reviewed).

Pathogen name	Climate Driver	Minimum score	Median score	Maximum score	PMIDs of papers
Acinetobacter baumannii	Moisture	60	70	80	11591541,8940416
Acinetobacter baumannii	Particle matter	20	50	80	15566039,18247474
Acinetobacter baumannii	Temperature	80	80	80	19031546
Aeromonas hydrophila	Extreme weather events	20	20	20	12113407
Aeromonas hydrophila	Moisture	20	20	20	10946412
Aeromonas hydrophila	Rainfall	20	20	20	8513068
Aeromonas hydrophila	Salinity	15	20	40	31839,16345850,15678554
Anaplasma phagocytophilum	Altitude	20	20	20	17993656
Anaplasma phagocytophilum	Climate change	40	40	40	18634803
Anaplasma phagocytophilum	Moisture	-20	20	40	18047394,18429692,18260527, 20618646
Anaplasma phagocytophilum	Rainfall	80	80	80	15857659,18429692
Anaplasma phagocytophilum	Temperature	40	40	40	18634803
Anaplasma phagocytophilum	Vegetation	40	40	40	18047394
Ascaris suum	Altitude	20	20	20	2787072
Aspergillus niger	Altitude	80	80	80	16822572
Aspergillus niger	Moisture	-40	20	80	6402980,16687184,16772229, 16891107
Aspergillus niger	Salinity	15	15	15	562476
Aspergillus niger	Wind	40	60	80	16772229,21328007
Bacillus anthracis	Climate change	40	40	40	20231894
Bacillus anthracis	Moisture	5	5	5	19720074
Bacillus anthracis	Particle matter	15	15	80	18487406,15078521,21835070,20193714,1693679
Bacillus anthracis	Rainfall	5	22.5	40	19720074,18165531
Bacillus anthracis	Temperature	5	5	5	19720074
Bacillus anthracis	Vegetation	5	5	5	19720074
Bacillus anthracis	Wind	-20	10	10	7973702,9633664,10475972
Bacillus cereus	Particle matter	20	20	20	17145080, 10212408
Bacillus licheniformis	Particle matter	20	20	20	18321601
Bacillus subtilis	Moisture	15	15	15	19040453

Bacteroides fragilis	Temperature	80	80	80	20851970
Bartonella henselae	Moisture	20	50	80	7561200,12680718
Bartonella henselae	Rainfall	80	80	80	7561200
**Bluetongue Virus**	Altitude	-10	80	80	18922155,17997043,15189233,11732415,15934613
**Bluetongue Virus**	Climate change	5	5	80	15685226,21151914,20167199,18799177,21570141
**Bluetongue Virus**	Moisture	-10	60	80	18922155,12109708,15189233,19493190,11732415
**Bluetongue Virus**	Temperature	40	80	80	20179768,17997043,11004778,14651652,20377730
**Bluetongue Virus**	Vegetation	40	40	80	17997043,15189233,18498611
**Bluetongue Virus**	Wind	5	20	40	19687037,19376658,18639355,18649960,21300413
Borrelia burgdorferi	Altitude	20	80	80	1951802,19685082,17417957,19492952,17695026
Borrelia burgdorferi	Climate change	5	20	20	15006781,19685082,18262840,18634803,19536444
Borrelia burgdorferi	Moisture	5	80	80	19685082,18262840,17417957,17203287,21175072
Borrelia burgdorferi	Oscillations	80	80	80	12680924
Borrelia burgdorferi	Rainfall	-80	80	80	16669698,9169184,15857659,12653136,15228815
Borrelia burgdorferi	Temperature	15	15	15	7790468
Borrelia burgdorferi	Vegetation	80	80	80	16606998
**Bovine Herpesvirus 1**	Altitude	80	80	80	21501883
**Bovine Herpesvirus 1**	Temperature	20	20	20	18304663
**Bovine Viral Diarrhea Virus 1**	Altitude	80	80	80	21822791
Brucella abortus	Moisture	20	20	20	7949352
Brucella abortus	Oscillations	5	5	5	12414142
Campylobacter coli	Temperature	15	60	60	9464397,10389249,11168729
Campylobacter jejuni	Moisture	60	60	60	16830853
Campylobacter jejuni	Rainfall	-20	20	80	20891037,20965083,6092418,6330161,14662945
**Canine Distemper Virus**	Rainfall	80	80	80	18575601
Chlamydia trachomatis	Altitude	80	80	80	18394663
Chlamydia trachomatis	Extreme weather events	40	40	40	18955385
Chlamydia trachomatis	Moisture	20	50	80	21829735,20608763
Chlamydia trachomatis	Rainfall	20	50	80	10227221,21829735
Chlamydophila abortus	Particle matter	20	20	20	20976071,18230187
Chlamydophila pneumoniae	Moisture	60	60	60	8368846
Chlamydophila psittaci	Particle matter	5	20	20	20976071,18977610,18230187
**Classical Swine Fever Virus**	Moisture	15	15	15	15158066
**Classical Swine Fever Virus**	Temperature	15	15	15	15158066,1335310
**Classical Swine Fever Virus**	Wind	-40	20	80	14623411,11601514
Clostridium botulinum	Particle matter	5	15	20	7005856,20004414,6400580,19831311
Clostridium botulinum	Wind	20	20	20	7005856
Clostridium difficile	Rainfall	20	20	20	2666437
Clostridium perfringens	Extreme weather events	20	20	80	15899275,17141293,18804835,11976088,12474967
Clostridium perfringens	Particle matter	20	80	80	21492899,14711629,21571877
Coxiella burnetii	Moisture	15	15	15	21563011

Coxiella burnetii	Particle matter	5	17.5	20	7671918,15437000,21571877,9525780
Coxiella burnetii	Rainfall	20	80	80	21810740,15324547,11443552
Coxiella burnetii	Wind	5	80	80	15324547,15324553,10400556,16284874,16481501
Cryptococcus neoformans	Moisture	20	50	80	15965721,10206749
Cryptococcus neoformans	Rainfall	-40	20	80	8648227,10468126
Cryptosporidium parvum	Extreme weather events	80	80	80	9790442
Cryptosporidium parvum	Particle matter	-40	60	80	17583766,18367230,19539587
Cryptosporidium parvum	Rainfall	15	60	80	8035045,18367230,20637489,12406745,16488039
Cryptosporidium parvum	Salinity	60	60	80	16672482,10628695,10799838,18026231
Cryptosporidium parvum	Temperature	15	80	80	16047784,8919806,8919806,16000797,20023095
Echinococcus granulosus	Climate change	5	5	5	21802208
Echinococcus granulosus	Extreme weather events	5	5	5	21802208
Echinococcus granulosus	Moisture	60	60	60	17933466
Echinococcus granulosus	Rainfall	5	20	80	2210938,20071084,21802208,2260900,20393798
Echinococcus granulosus	Temperature	5	5	5	21802208
Encephalitozoon cuniculi	Salinity	15	15	15	15270118
**Encephalomyocarditis Virus**	Moisture	15	15	15	166928
Entamoeba histolytica	Climate change	5	5	5	15567581
Entamoeba histolytica	Moisture	60	60	60	12751714
Entamoeba histolytica	Rainfall	20	20	80	226490,202045,19248646
Enterobacter aerogenes	Temperature	15	15	15	1100601
Enterobacter cloacae	Particle matter	15	15	15	17655192
Enterococcus faecalis	Salinity	60	60	60	20864199
Enterococcus faecium	Temperature	15	15	15	6841320
Escherichia coli	Altitude	80	80	80	16118577
Escherichia coli	Extreme weather events	20	40	80	16022790,10401868,11581467,16913133,16955897
Escherichia coli	Wind	80	80	80	12966970,18605570,19320169,16310242,18242661
***Escherichia coli* O157:h7**	Extreme weather events	5	12.5	20	18174909,15371222
***Escherichia coli* O157:h7**	Rainfall	80	80	80	18174909
Fasciola hepatica	Altitude	5	20	80	16150452,10444322,11769277,18205983,20071083
Fasciola hepatica	Climate change	10	20	20	21249228,19556065,21493511
Fasciola hepatica	Extreme weather events	10	20	20	17933308,21493511,3631704
Fasciola hepatica	Moisture	20	40	80	9735915,9735916,15110406,15682338,20464681
Fasciola hepatica	Rainfall	20	40	80	21249228,20227416,20887726,21055879,21111536
Fasciola hepatica	Temperature	-40	20	80	10444322,9735915,20071083,2050981,978356
Fasciola hepatica	Vegetation	40	40	40	9735915
**Foot-and-Mouth Disease Virus**	Moisture	15	15	15	4337217
**Foot-and-Mouth Disease Virus**	Wind	20	20	20	12002549,12002550

Francisella tularensis	Rainfall	40	60	80	14620945,18385353
Francisella tularensis	Temperature	80	80	80	14620945
Fusarium oxysporum	Particle matter	20	20	20	20820862,3935928,2938009,1702081
**Gallid Herpesvirus 2**	Particle matter	20	20	20	16290211,16678918,18770332
Gibberella moniliformis	Moisture	-80	20	80	2942448,15487329,20549795
Gibberella moniliformis	Rainfall	20	20	60	20491926,11789932,2942448,20127546,19003428
Gibberella moniliformis	Temperature	-15	37.5	60	18263743,20491926,15487329,8890481
Haemonchus contortus	Climate change	20	30	40	18824303,19556065
Haemonchus contortus	Extreme weather events	15	15	15	8220500
Haemonchus contortus	Moisture	-20	80	80	1399249,1428508,9668516,18824304,20149541
Haemonchus contortus	Rainfall	15	20	20	2777463,2332275,8220500,9668516,9668516
Haemonchus contortus	Temperature	5	20	80	1428508,8447070,15817209,17011129,16336722
Helicobacter pylori	Altitude	-40	10	80	1612475,15810948,21083747,8284633,9218856
Helicobacter pylori	Rainfall	-40	20	80	18509453,16151096
**Hepatitis A Virus**	Extreme weather events	20	30	40	17306323,17037154
**Hepatitis A Virus**	Oscillations	20	20	20	15115435
**Hepatitis A Virus**	Rainfall	20	20	20	16980430
**Hepatitis B Virus**	Rainfall	20	20	20	9447780
**Hepatitis E Virus**	Extreme weather events	-20	20	20	21443914,10492753,12497976,9311635,11003732
**Hepatitis E Virus**	Rainfall	-20	20	80	20801864,17935174,7660427,10492753,12497976
**Human Enterovirus C**	Moisture	10	15	15	44178,6257161,2984990,20025904
**Human Enterovirus C**	Salinity	15	15	15	184736
**Human Herpesvirus 3**	Moisture	20	20	20	6519892
**Human T-Lymphotropic Virus 1**	Altitude	20	20	20	2777408,10752214
**Influenza A Virus**	Climate change	5	5	5	18819672
**Influenza A Virus**	Moisture	-80	60	80	18881494,17953482,19663691,20879867,19635124
**Influenza A Virus**	Particle matter	10	15	60	20435545,17297505,21111917
**Influenza A Virus**	Rainfall	-80	40	80	19635124,8490980,18509468
**Influenza A Virus**	Salinity	15	15	60	2142421,17494568,20521724
**Influenza A Virus**	Temperature	40	40	40	20386716
Klebsiella oxytoca	Particle matter	20	20	20	10212408,7820386
Klebsiella pneumoniae	Moisture	-40	27.5	40	18260762,18260762,21966489,1499666
Legionella pneumophila	Particle matter	80	80	80	19120656,10281778
Legionella pneumophila	Rainfall	20	20	20	19751596,8285669
Legionella pneumophila	Wind	15	15	15	18939571
Leishmania donovani	Altitude	20	20	20	20503185
Leishmania donovani	Moisture	20	20	20	20503185
Leishmania infantum	Altitude	20	80	80	21854702,18986768,9879555
Leishmania infantum	Moisture	80	80	80	20816927,17017238
Leishmania infantum	Rainfall	20	80	80	11127239,15975718,20377867,8667372,17017238

Leishmania infantum	Temperature	20	40	80	19271999,20816927,20377867,21080321
Leishmania infantum	Wind	80	80	80	17017238
Listeria monocytogenes	Extreme weather events	20	20	20	7259653
Listeria monocytogenes	Particle matter	20	20	80	21889781,18321601,18939743
Listeria monocytogenes	Rainfall	60	70	80	19339033,21889781
Listeria monocytogenes	Salinity	-20	15	20	2117874,16371177,18592742
Listeria monocytogenes	Temperature	80	80	80	21889781
Mannheimia haemolytica	Moisture	60	60	60	2138372,3417911
Mannheimia haemolytica	Particle matter	45	45	45	12567239
Mannheimia haemolytica	Temperature	60	60	60	2138372
Moraxella catarrhalis	Rainfall	40	40	40	18162931
Mycobacterium avium	Altitude	80	80	80	12680607
Mycobacterium avium	Particle matter	20	20	80	16349305,11229914,20656861,20036081,21610001
Mycobacterium avium	Rainfall	15	40	60	16751517,21239557,17083748
Mycobacterium avium* subsp. *paratuberculosis	Particle matter	80	80	80	20656861,20036081,21610001
Mycobacterium avium* subsp. *paratuberculosis	Rainfall	80	80	80	16751517,16751517
Mycobacterium bovis	Altitude	80	80	80	16032168
Mycobacterium bovis	Extreme weather events	80	80	80	16618553
Mycobacterium tuberculosis	Altitude	80	80	80	17880701,12887034,16837708
Mycoplasma pneumoniae	Moisture	-80	15	40	19318345,5686020,5808076,21822990
Mycoplasma pneumoniae	Temperature	80	80	80	21822990
**Myxoma Virus**	Wind	10	10	10	2881798
Neisseria meningitides	Moisture	40	80	80	19164421,18983283,12141615
Neisseria meningitides	Particle matter	80	80	80	21511508
Neospora caninum	Moisture	80	80	80	19210662
Neospora caninum	Rainfall	-80	80	80	14636679,15740859,19210662,21898183
Neospora caninum	Temperature	-80	80	80	14636679,15471005,15740859
**Newcastle Disease Virus**	Particle matter	80	80	80	5963856
Pasteurella multocida	Moisture	20	40	60	1575496,7373722
Pasteurella multocida	Particle matter	45	45	45	12567239
**Porcine Circovirus**	Particle matter	80	80	80	19773132
**Porcine Reproductive and Respiratory Syndrome Virus**	Moisture	60	70	80	17156739,20667494
**Porcine Reproductive and Respiratory Syndrome Virus**	Temperature	60	60	60	17156739,20224088
Porphyromonas gingivalis	Temperature	15	60	60	7927742,10085036,11014869
Pseudomonas aeruginosa	Moisture	10	15	20	21966489,14368472
**Rabies Virus**	Altitude	20	20	20	17085890
**Rabies Virus**	Rainfall	20	50	80	3999245,17255450
**Respiratory Syncytial Virus**	Altitude	5	10	40	3198947,16452353,12671449
**Respiratory Syncytial Virus**	Moisture	-10	40	40	19663691,19635124,11864206,15921045,20118684

**Respiratory Syncytial Virus**	Rainfall	-80	20	80	19635124,11864206,21865418,16678480,22001966
**Respiratory Syncytial Virus**	Wind	-10	15	40	11864206,21044325
**Rotavirus A**	Extreme weather events	20	20	20	1653263
**Rotavirus A**	Moisture	20	20	20	15022939
**Rotavirus A**	Particle matter	20	20	20	2557702
**Rotavirus A**	Rainfall	20	20	80	20684698,20684712,2853177,1653263,17385670
Salmonella enterica* subsp. *enterica enteritidis	Particle matter	-20	20	80	12558590,8810013,9355251,19237216,21035883
Salmonella enterica* subsp. *enterica typhimurium	Moisture	60	60	60	19054240
Salmonella enterica* subsp. *enterica typhimurium	Particle matter	80	80	80	17012158
Salmonella enterica* subsp. *enterica typhimurium	Rainfall	20	20	20	18444810
Salmonella enterica* subsp. *enterica typhimurium	Temperature	20	20	20	12039728
**SARS Coronavirus**	Temperature	5	5	5	15035025
Serratia marcescens	Temperature	20	20	20	12077296
Shigella dysenteriae	Rainfall	10	20	20	1099145,9463662,2200700
Shigella flexneri	Extreme weather events	80	80	80	17602851
Shigella flexneri	Rainfall	20	20	20	2200700,1452309
Staphylococcus aureus	Extreme weather events	20	20	20	15891349
Staphylococcus aureus	Moisture	-40	20	60	21966489,11916706,14612371
Staphylococcus aureus	Salinity	80	80	80	21917291
Staphylococcus epidermidis	Particle matter	20	20	20	18321601
Streptococcus pneumoniae	Climate change	80	80	80	19961583
Streptococcus pneumoniae	Moisture	10	10	10	21553207
Streptococcus pneumoniae	Rainfall	10	15	20	10913396,10427935
Streptococcus pneumoniae	Temperature	80	80	80	8824973
Streptococcus pneumoniae	Wind	60	60	60	17126867
**Suid Herpesvirus 1**	Moisture	60	60	60	2156471
**Suid Herpesvirus 1**	Wind	60	60	60	1659567
Toxoplasma gondii	Altitude	80	80	80	18801208,16280199,19246158,20096148,9658447
Toxoplasma gondii	Climate change	5	5	5	19418068
Toxoplasma gondii	Extreme weather events	20	20	20	21281213
Toxoplasma gondii	Moisture	20	60	60	18316161,7431517,16539014
Toxoplasma gondii	Rainfall	5	80	80	12076629,16157341,16989836,18452923,19619946
**Transmissible Gastroenteritis Virus**	Moisture	60	60	60	20228108
**Transmissible Gastroenteritis Virus**	Temperature	60	60	60	20228108
Trichinella spiralis	Altitude	80	80	80	8939049
Trichinella spiralis	Moisture	5	5	5	11099840
Trichostrongylus colubriformis	Moisture	-60	45	60	2332277,4661479,7201628,2800302,17152941
Trichostrongylus colubriformis	Rainfall	20	20	20	2332275,2777463,7735304
Vibrio cholera	Climate change	5	5	5	12364378,1820262

Vibrio cholera	Extreme weather events	10	80	80	16760521,17123999,18981509,12452126,21537092
Vibrio cholera	Moisture	5	22.5	40	14379012,21415487
Vibrio cholera	Oscillations	5	15	60	8953025,10865299,15186348,18793311,11220769
Vibrio cholera	Particle matter	80	80	80	21642406,18545963
Vibrio cholera	Rainfall	5	20	40	19001267,8225749,7148820,1294392,3038946
Vibrio cholera	Salinity	5	80	80	12364378,16085859,21642406
Vibrio cholera	Temperature	15	20	80	10865299,1294392,2764569,12732548,14602587
Vibrio cholera	Wind	40	40	40	17384764
Vibrio parahaemolyticus	Extreme weather events	20	20	20	18697592
Vibrio parahaemolyticus	Oscillations	5	20	40	18854707,12462995,6702183
Vibrio parahaemolyticus	Particle matter	80	80	80	20100246,17921270,20817802,18963158,19732825
Vibrio parahaemolyticus	Rainfall	20	20	20	1294392,9065272,6370564,11248518,22014235
Vibrio parahaemolyticus	Salinity	-20	20	80	11000648,20100246,4549489,3183022,367273
Vibrio parahaemolyticus	Temperature	15	20	80	20100246,1294392,19930458
**West Nile Virus**	Climate change	-5	5	80	18799177,20181272,19580695,19412377,11419587
**West Nile Virus**	Extreme weather events	-10	20	80	17479899,17953117,18439367,8835346,11192289
**West Nile Virus**	Moisture	40	40	80	19654911,18279008,19664833,16599151,16507476
**West Nile Virus**	Rainfall	-40	40	80	19654911,19057643,16459215,21802710,18799177
**West Nile Virus**	Temperature	5	40	60	18584026,19654911,16459215,19406093,18279008
**West Nile Virus**	Vegetation	40	40	60	16011435,19054585,19440960
Yersinia pestis	Climate change	20	20	20	16924109
Yersinia pestis	Oscillations	-40	40	40	18765356,10586917,20810830,20810830,11340502
Yersinia pestis	Rainfall	5	20	80	11927030,19584125,6364800,13115987,18197776
Yersinia pseudotuberculosis	Extreme weather events	20	20	20	3284516
Yersinia pseudotuberculosis	Rainfall	10	20	20	7779969,16030939,3284516

Table S7. Results (P values) of Fisher's Exact test comparisons of the frequency of positive median evidence for sensitivity to climate drivers (0, 1, 2, 3, 4+) compared to (a) the taxonomic division of pathogens, and (b) the transmission routes of pathogens. P values in bold show significant ($P<0.05$) differences.

(a)		Bacteria	Fungi	Helminths	Protozoa
	**Fungi**	0.234			
	**Helminths**	**0.030**	**0.012**		
	**Protozoa**	**0.005**	0.072	0.344	
	**Viruses**	**0.045**	0.382	**0.001**	**0.002**

(b)		dcont [a]	sex [a]	soil [a]	water [a]	air [a]	fomite [a]	vector [a]
	**sex**	0.817						
	**soil**	0.353	0.437					
	**water**	**0.001**	**0.007**	0.498				
	**air**	0.739	0.431	0.600	**0.026**			
	**fomite**	0.993	0.865	0.519	**0.005**	0.822		
	**vector**	**<0.001**	**0.010**	0.813	0.191	**0.011**	**0.004**	
	**food**	**0.028**	0.171	0.918	0.407	0.176	0.100	0.190

[a] Transmission routes include: airborne (via inhalation) (air), direct (non-sexual) contact (dcont), fomite (fomite), foodborne (via ingestion) (food), direct sexual contact - (sex), soil-borne (soil), vector-borne (vector) and waterborne (water).

References

1 McIntyre, K. M., Hawkes, I., Waret-Szkuta, A., Morand, S. & Baylis, M. The H-Index as a Quantitative Indicator of the Relative Impact of Human Diseases. *PLoS One* **6**, e1955810. doi: 1371/journal.pone.0019558 (2011).

2 McIntyre, K. M. *et al.* A Quantitative Prioritisation of Human and Domestic Animal Pathogens in Europe. *PLoS One* **9**, e103529. doi:10.1371/journal.pone.0103529 (2014).

3 McIntyre, K. M. *et al.* Using open-access taxonomic and spatial information to create a comprehensive database for the study of Mammalian and avian livestock and pet infections. *Prev. Vet. Med.* **116**, 325-335, doi:10.1016/j.prevetmed.2013.07.002 (2014).

4 Wardeh, M., Risley, C., McIntyre, M. K., Setzkorn, C. & Baylis, M. Database of host-pathogen and related species interactions, and their global distribution. *Sci. Data* **2**, 150049, doi:10.1038/sdata.2015.49 (2015).

5 National Center for Biotechnology Information, "PubMed" (www.ncbi.nlm.nih.gov/pubmed/) (U.S. National Library of Medicine, Bethesda, Maryland, USA).

6 National Center for Biotechnology Information, "The NCBI Taxonomy database homepage" (www.ncbi.nlm.nih.gov/Taxonomy/) (U.S. National Library of Medicine, Bethesda, Maryland, USA).

7 International Committee on Taxonomy of Viruses (ICTV). (www.ictvdb.org/Ictv/index.htm). (2012).

8 Aitken, I. *Diseases of sheep* (Blackwell, Oxford, UK, 2007).

9 Calnek, B. W. , Barnes, H. J., Beard, C. W. & McDougald, L. R. (eds.) *Diseases of poultry* (Wiley, 1997).

10 Coetzer, J. A. W. & Tustin, R. C. *Infectious diseases of livestock* (OUP Southern Africa, 2005).

11 Green, C. E. *Infectious diseases of the dog and cat* (Elsevier Saunders, 2012).

12 Harcourt-Brown, F. *Textbook of rabbit medicine* (Butterworth-Heinemann, 2001).

13 Magill, A. J., Ryan, E. T., Solomon, T. & Hill, D. R. *Hunter's tropical medicine and emerging infectious disease* (Elsevier Saunders, 2012).

14 Mandell, G. l., Bennett, J. D. & Dolin, R. *Principles and practice of infectious diseases* (Churchill Livingstone, 2009).

15 Straw, B. E. *Diseases of swine* (Wiley-Blackwell, 2006).

16 Taylor, L. H., Latham, S. M. & Woolhouse, M. E. J. Risk factors for human disease emergence. *Philos. T. Roy. Soc. B* **356**, 983-989 (2001).

17 Woolhouse, M. E. J. & Gowtage-Sequeria, S. Host range and emerging and reemerging pathogens. *Emerg. Infect. Dis.* **11**, 1842-1847 (2005).

18 Jones, K. E. *et al.* Global trends in emerging infectious diseases. *Nature* **451**, 990-U994, doi:10.1038/nature06536 (2008).

19 R Core Team. R: A Language and Environment for Statistical Computing v. Version 3.0.1 (2013-05-16) - "Good Sport" (Copyright (C) 2013 The R Foundation for Statistical Computing, Vienna, Austria, 2013).
